# Impact of Alpha-Lipoic Acid Chronic Discontinuous Treatment in Cardiometabolic Disorders and Oxidative Stress Induced by Fructose Intake in Rats

**DOI:** 10.3390/antiox8120636

**Published:** 2019-12-11

**Authors:** Steliana Ghibu, Cristina Elena Craciun, Razvan Rusu, Claudiu Morgovan, Cristina Mogosan, Luc Rochette, Adrian Florin Gal, Maria Dronca

**Affiliations:** 1 Department of Pharmacology, Physiology and Pathophysiology, Faculty of Pharmacy, “Iuliu Haţieganu” University of Medicine and Pharmacy, 400349 Cluj-Napoca, Romania; stelianaghibu@yahoo.com; 2 Department of Pharmaceutical Biochemistry and Clinical Laboratory, Faculty of Pharmacy, “Iuliu Haţieganu” University of Medicine and Pharmacy, 400349 Cluj-Napoca, Romania; ecgagyi@yahoo.com; 3 Department of Medical Biochemistry, Faculty of Medicine, “Iuliu Haţieganu” University of Medicine and Pharmacy, 400349 Cluj-Napoca, Romania; imunorusu@yahoo.com (R.R.); m_dronca@yahoo.com (M.D.); 4 Preclinical Department, “Lucian Blaga” University of Sibiu, 550169 Sibiu, Romania; claudiumorgovan@yahoo.com; 5 Equipe d’Accueil (EA 7460), Physiopathologie et Epidémiologie Cérébro-Cardiovasculaires (PEC2), Université de Bourgogne - Franche Comté, Faculté des Sciences de Santé, 7 Bd Jeanne d’Arc, 21000 Dijon, France; 6 Department of Cell Biology, Histology and Embryology, Faculty of Veterinary Medicine, University of Agricultural Sciences and Veterinary Medicine, 400372 Cluj-Napoca, Romania; adrian.gal@usamvcluj.ro

**Keywords:** alpha-lipoic acid, antioxidants, fructose-enriched diet, insulin resistance, hypertension, oxidative stress

## Abstract

Insulin resistance (IR) and cardiometabolic disorders are the main consequences of today’s alimentary behavior. This study evaluates the effects of a chronic-discontinuous treatment with alpha-lipoic acid (AL), an antioxidant substance that improves glycemic control associated with diabetes mellitus, on metabolic disorders and plasma oxidative stress induced by fructose intake, in rats. Sprague-Dawley rats (48 animals) were randomized into two series (*n* = 24): rats fed with standard chow or with standard chow supplemented with 60% fructose. In each of the two series, for 2 weeks/month over 12 weeks, a group of rats (*n* = 12) was intraperitoneally injected with NaCl 0.9%, and a second group (*n* = 12) received AL 50 mg/kg/day. Body weight, glycemia, and systolic blood pressure were monitored throughout the study. After 12 weeks, IR, plasma lipoproteins, uric acid, transaminase activities, and oxidative stress markers were assessed. The high fructose-enriched diet induced cardiometabolic disorders (hypertension, hyperglycemia, IR and dyslipidemia), an increase in uric acid concentration, transaminase activities and C-reactive protein level. This diet also enhanced plasma products of lipid and protein oxidation, homocysteine level, and decreased GSH/GSSG ratio. In this field, there is evidence to indicate that oxidative stress plays an important role in the etiology of diabetic complications. AL discontinuous treatment prevents the metabolic disorders induced by fructose intake, reduced plasma lipid and protein oxidation-products, and restored the GHS/GSSG ratio. Our study proves a promising potential of the chronic-discontinuous treatment of AL and highlights the pleiotropic effects of this antioxidant substance in metabolic disorders such as diabetes.

## 1. Introduction

Obesity and insulin resistance (IR) are the main consequences of today’s alimentary behavior developed on the background of a high-carbohydrates and high-fat diet consumption. The IR, characterized by hyperglycemia and compensatory hyperinsulinemia—as consequence of the impairment of the insulin signaling pathway—represents the main feature of metabolic syndrome and type 2 diabetes mellitus, and a high-risk factor for cardiovascular diseases as well [1,2]. This metabolic disturbance has attracted attention during the past decades because of its high prevalence, particularly in young population, and because of its long-term consequences: alteration of lipid metabolism (dyslipidemia), endothelial dysfunction and hypertension, the development of a vascular and general inflammatory state, and the onset of atherosclerosis [1,3]. 

Besides genetic susceptibility, hypercaloric diet intake, or obesity [4], oxidative stress (OS) plays an important role in the pathogenesis of IR [5,6,7]. On the other hand, hyperglycemia and IR, dyslipidemia, and hypertension are characterized by a significant production of reactive oxygen species (ROS) and by an impairment of endogenous antioxidant systems [8], thus creating a vicious circle which amplifies the pre-existing disorders [9,10]. Also, in this field, there is evidence to indicate that oxidative stress plays an important role in the etiology of diabetic complications [11]. Therefore, the relationship between OS and IR could be multifactorial [6,11]. Under these circumstances, the superoxide anion (O_2_•^−^), the first step in the ROS synthesis could be formed in excess by mitochondrial dysfunction, by plasma glucose oxidation and lipid peroxidation, or through the activation of some vascular enzymes: NADPH oxidases, “uncoupled” endothelial nitric oxide synthase (eNOS), xanthine oxidase (XO), and cyclooxygenases (COX) [9,10,11]. 

To restore the tissue sensitivity to insulin and to prevent the long-term IR complications, lifestyle change (caloric restriction, regular physical activity) [12] and specific drug therapy are recommended [13]. Considering that there are only a few insulin-sensitizing drugs (e.g., metformin, pioglitazone) [1,14,15] and that the OS plays an important role in the onset and maintenance of IR [7], it seems interesting to evaluate the efficacy of the antioxidants in this metabolic disorder. 

An antioxidant is defined as “any substance that, when present at low concentrations compared with those of an oxidizable substrate (every organic molecule found in vivo) significantly delays or prevents oxidation of that substrate” [16]. Given the variety of antioxidant agents, an antioxidant substance that can interfere at multiple levels or a combination of different antioxidants deserves higher attention than a conventional antioxidant substance. In this context, the alpha-lipoic acid (AL) or thioctic acid (6,8-dithio-octanoic) seems to be the right candidate. As an antioxidant, AL is able to scavenge reactive oxygen species (HO•, HClO, ^1^O_2_), chelate transition metals (iron and copper), or regenerate reduced forms of some antioxidants (vitamin E, vitamin C, and glutathione) [17]. A small amount of AL is synthesized de novo from fatty acids and cysteine but the most part of the AL remains to be absorbed from exogenous sources [18]. Exogenous AL is quickly absorbed and, due to its amphiphilic properties, it is widely distributed in cellular and extracellular environments [19]. In some European countries, AL has been used for years as adjuvant therapy for diabetic neuropathy being frequently recommended as a long-term discontinuous treatment. In addition, some of its benefits were reported in several experimental studies [20,21,22,23] in which the metabolic disorders were induced by using food similar to that responsible for the development of human IR. Nevertheless, in most previous studies, the AL intake was begun simultaneously with the specific diet, being a preventive treatment, or it was administered continuously over a long period. Both of these forms of treatment are difficult to recommend and apply in humans. 

In this context, the aim of our study was to assess the impact of a chronic discontinuous treatment with AL in the fructose-fed rats, once the vascular and metabolic disturbances had been installed.

## 2. Materials and Methods 

### 2.1. Animals and Experimental Protocol

This study was approved by the Ethics Committee of the “Iuliu Hatieganu” University of Medicine and Pharmacy, Cluj-Napoca, Romania (no. 237/31.05.2018) and was performed in accordance with EU Directive 2010/63/EU for animal experiments. 

**Figure 1:**
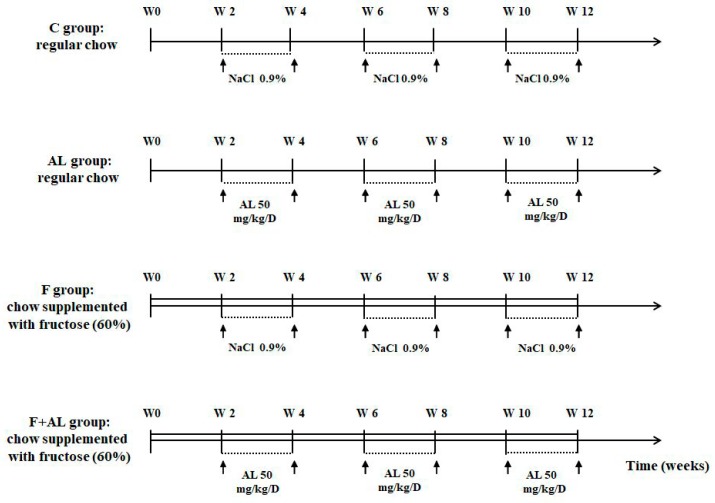
Experimental protocol. In the control group (C, *n* = 12), rats received regular chow and were intraperitoneally (i.p.) injected with saline solution for 2 weeks/month over 12 weeks. In the lipoic acid-treated group (AL, *n* = 12), rats received regular chow and were injected i.p. with 50 mg/kg/day alpha-lipoic acid for 2 weeks/month during 12 weeks. In the fructose-fed group (F, *n* = 12), rats received chow supplemented with fructose (60%) and were injected i.p. with saline solution for 2 weeks/month over 12 weeks. In the fructose-fed group treated with alpha-lipoic acid (F + AL, *n* = 12), rats received chow supplemented with fructose (60%) and were injected with the same dose of alpha-lipoic acid as the previous group. The rats were sacrificed after 12 weeks of diet and chronic discontinuous treatment.

Forty-eight male Sprague-Dawley rats (10 weeks old and weighing 200–250 g) had access to water and specific chow *ad libitum* and were randomly divided into 4 groups, organized in 2 series: rats fed with regular chow, and those on a fructose-enriched diet, respectively. Each group of rats was intraperitoneally (i.p.) injected for 2 weeks/month (W3-W4, W7-W8, and W11-W12) over 12 weeks (Figure 1), as follows:

- Control group (C, *n* = 12) received regular chow and the rats were injected i.p. with saline solution (NaCl 0.9%).
- Lipoic acid-treated group (AL, *n* = 12) received regular chow and the rats were injected i.p. with 50 mg/kg/day racemic alpha-lipoic acid (Thiogamma 600^®^, Wörwarg Pharma, Germany) for 14 days/month over 12 weeks.
- Fructose-fed group (F, *n* = 12) received chow supplemented with fructose (60%) and the rats were injected i.p. with saline solution.
- Fructose-fed group treated with alpha-lipoic acid (F + AL, *n* = 12) received chow supplemented with fructose (60%) and the rats were injected with the same dose of alpha-lipoic acid at the same intervals of times as the previous group (AL group).

The Sprague-Dawley rats were purchased from the “Cantacuzino” National Institute of Research-Development for Microbiology and Immunology, Bucharest, Romania and the specific food for rats from Ssniff^®^, Soest, Germany. The high-fructose diet (Ssniff^®^, Soest, Germany) was composed of 60% fructose, 20% casein, 6.50% cellulose powder, 6% fat, 6% minerals & trace element premix, 1% vitamin premix, 0.30% l-Cystine, and 0.20% Choline chloride. 

The body weight of rats was monitored every 7 days. After 12 weeks from the beginning of the study, the rats were fasted overnight, were anesthetized with sodium thiopental (50 mg/kg body weight, i.p.) and the blood was collected by retro-orbital sinus puncture. Integral blood, plasma, and serum were stored at −80 °C until analyses were performed. 

### 2.2. Hemodynamic Parameters 

Systolic blood pressure (SBP) and heart rate (HR) were evaluated in the conscious rats every 2 weeks by a non-invasive tail-cuff technique (58500—Ugo Basile blood pressure recorder coupled with a rodent heater, Hugo Basile, Italy). The rats were handled repeatedly and were allowed to adapt to the restraint chamber before the beginning of the study and during the entire period of the study, between the different measurements. Before each measurement the rats were pre-warmed at 30 °C for a minimum of 30 min. The mean of six consecutive readings was recorded as the individual blood pressure value. 

### 2.3. Biochemical Investigations 

The fasting glycemia (mg/dL) was evaluated every 2 weeks by a glucometer (Accu-Chek, Roche, Mannheim, Germany). At the end of the study, serum fasting glucose (mg/dL) was measured with the glucose oxidase colorimetric kit (Glucose GOD/PAP; Diagnosticum ZRT, Budapest, Hungary) and insulin (μU/mL) was assessed using the insulin (rat) ultrasensitive ELISA kit (ALPCO Diagnostics, Salem, NH, USA). Insulin resistance was estimated by the homeostasis model assessment of insulin resistance (HOMA-IR) defined as fasting glucose (mg/dL) × insulin (μU/mL)/405 [24,25]. 

The other serum biochemical markers were performed with spectrophotometric methods, using commercially available kits and a Konelab 20i automatic analyzer (Thermo SCIENTIFIC, Vantaa, Finland). The total cholesterol, low-density lipoprotein cholesterol (LDL-C), and C-reactive protein (CRP) were determined using the kits from Thermo SCIENTIFIC (Vantaa, Finland). The levels of triglycerides (TG), uric acid, and the activities of alanine aminotransferase (ALAT) and of aspartate aminotransferase (ASAT) were measured with specific kits from Diagnosticum ZRT, Budapest, Hungary.

### 2.4. Oxidative Stress Markers 

#### 2.4.1. Plasma Lipid and Protein Oxidation Products 

Malondialdehyde (MDA, μmol/L), the end-product of lipid peroxidation, was assessed by using a Chromsystems kit (Chromsystems Instruments and Chemicals GmbH, Munich, Germany), on Agilent-1100 HPLC system provided with fluorescent detection (Agilent Technologies; Wilmington, DE, USA), according to the supplier’s recommendation.

Protein carbonyl content (nmol/mg protein), an index of protein oxidative injury, was determined according to the spectrophotometric method of Reznick [26]. The protein content was measured by the Bradford method [27]. 

#### 2.4.2. Homocysteine

Total serum Homocysteine (Hcy, μmol/L), a marker of cardiovascular disease and oxidative stress, was determined by reverse-phase high performance liquid chromatography (RP-HPLC) with isocratic elution and fluorescence detection by using an Agilent chromatographic system and Chromsystems kit (Chromsystems Instruments and Chemicals GmbH, Munich, Germany).

#### 2.4.3. Circulating Antioxidants Levels 

Whole blood glutathione, reduced glutathione (GSH) and oxidized glutathione (GSSG), expressed as μmol/L, were assessed on Agilent-1100 HPLC system (Agilent Technologies; Wilmington, DE, USA) with fluorescence detection, following the supplier’s recommendation of the Chromsystems kit (Chromsystems Instruments and Chemicals GmbH, Munich, Germany). 

Glutathione peroxidase (GPx) activity, expressed as U/gHb, was determined in whole blood using a RANSEL kit (Randox Labs, Crumlin, UK) and a Cobas Mira Plus (Roche, Basel, Switzerland) analyzer. 

### 2.5. Histopathological Analysis

Liver tissue samples were withdrawn for further histological analysis. For the histopathologic investigation of the liver, the harvested samples were fixed in neutral 10% buffered formalin and were subsequently embedded in paraffin, sectioned at 4 μm thicknesses (Leica rotary microtome, RM2125, Nussloch, Germany) and stained with hematoxylin and eosin (H&E) and PAS methods (Periodic acid–Schiff staining). Histologic examination was performed with the aid of an Olympus BX51 microscope connected to a digital camera (Olympus DP-25, Tokyo, Japan). The microphotographs were acquired using an Olympus system for image acquisition and analysis (Olympus Cell B software, Tokyo, Japan).

### 2.6. Statistical Analysis 

All data are expressed as mean ± S.E.M. To compare the four groups at the end of the study, statistical analyses were performed with the one-factor analysis of variance (ANOVA) test; ANOVA was followed when necessary by a Newman–Keuls test. The body weight at the end of the study and the plasma insulin concentration were evaluated by a Student’s *t*-test. In order to compare the evolution of body weight, glycemia, and systolic blood pressure under diet and treatment, we used the two-factor repeated measures analysis of variance (ANOVA) test. Significance was established at a value of *p* < 0.05.

## 3. Results

### 3.1. Body Weight and Hemodynamic Parameters

The four groups of rats gained weight during the 12 weeks of study, but in our experimental conditions the fructose intake did not induce the increase in rats’ body weight. However, at the end of the study (W12), a significant weight loss was observed in both groups of rats treated with alpha-lipoic acid: AL and F + AL groups (Table 1). Nevertheless, there were no significant differences between the four groups of rats regarding the food and water consumption for the 12 weeks. 

**Figure 2:**
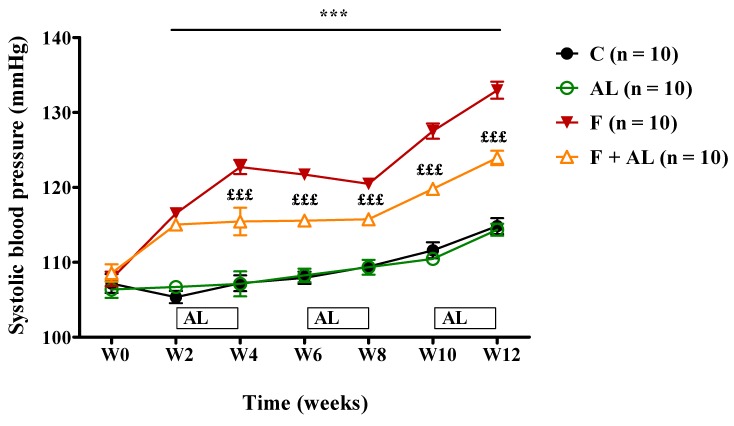
Evolution of the systolic blood pressure (SBP), assessed over the 12 weeks of the study in the control group (C), the alpha-lipoic acid-treated group (AL), the fructose-fed group (F), and the fructose-fed group treated with alpha-lipoic acid (F + AL) (^***^ *p* < 0.001: F and F + AL vs. C and AL; ^£££^ *p* < 0.001: F + AL vs. F).

A significant increase in the SBP was recorded starting with the second week, in the two groups of rats fed with fructose-enriched diet. This increase in the SBP was maintained for the next 10 weeks of the study. In fructose-fed rats, AL treatment attenuated the rise in SBP since the beginning of the treatment (W4) until the end of the study (Figure 2). No significant difference was observed in the HR between the four groups of rats throughout the experiment.

### 3.2. Biochemical Investigations 

**Figure 3:**
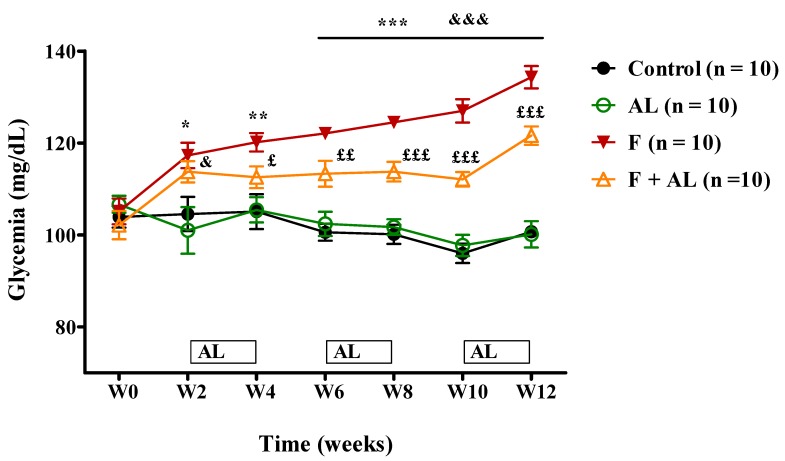
Evolution of the glycemia, assessed over the 12 weeks of the study in the Control group (C), the alpha-lipoic acid-treated group (AL), the fructose-fed group (F), and the fructose-fed group treated with alpha-lipoic acid (F + AL) (^*^ *p* < 0.05, ^**^ *p* < 0.01, ^***^ *p* < 0.001: F vs. C and AL; ^&^ *p* < 0.05, ^&&&^ *p* < 0.001: F + AL vs. C and AL; ^£^ *p* < 0.05, ^££^ *p* < 0.01, ^£££^ *p* < 0.001: F + AL *vs* F).

The fructose intake increased glycemia from the second week until the end of the study (Figure 3). Plasma insulin concentration and peripheral tissue resistance to insulin (HOMA-IR), assessed at the end of the study, had also been significantly increased (Table 1). The alpha-lipoic acid discontinuous treatment associated with the fructose-enriched diet reduced the rats’ glycemia (Figure 3) and plasma insulin concentration, and it improved the tissue sensitivity to insulin (Table 1). However, the treatment did not influence these parameters in the rats fed with standard chow (AL group) (Figure 3, Table 1).

At the end of the study, the fructose group presented a robust increase in serum total cholesterol (~1.4 fold), triglycerides (~5 fold), and LDL-cholesterol (~7 fold), parameters that were significantly reduced by alpha-lipoic acid discontinuous treatment (F + AL group, Table 2). 

**Figure 4:**
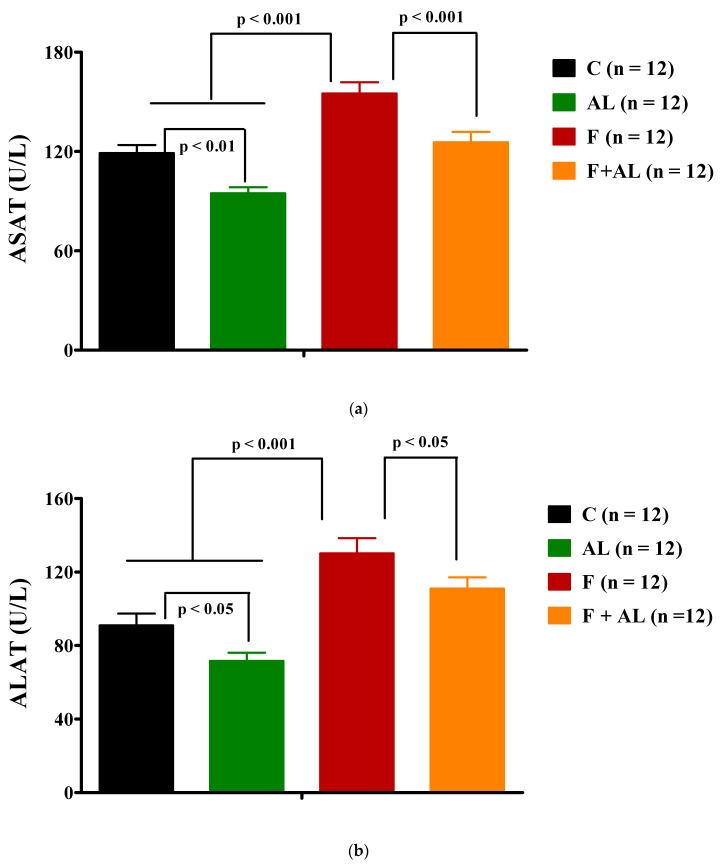
Transaminase activities: (**a**) Aspartate aminotransferase (ASAT) activity and (**b**) Alanine aminotransferase (ALAT) activity determined 12 weeks after diet and treatment in the control group (C), the alpha-lipoic acid-treated group (AL), the fructose-fed group (F), and the fructose-fed group treated with alpha-lipoic acid (F + AL).

Furthermore, after 12 weeks of fructose consumption the serum uric acid (Table 2), transaminase activities (ASAT and ALAT; Figure 4), and C-reactive protein level (Table 2) were found to be significantly increased in the F group, while in the F + AL group the values of these parameters were significantly reduced. On the other hand, the lipoic acid has induced a decrease in serum transaminase activities (Figure 4) and uric acid level (Table 2), even when it was associated with the regular diet (AL group).

### 3.3. Oxidative Stress Markers 

#### 3.3.1. Plasma Lipid and Protein Oxidation Products 

**Figure 5:**
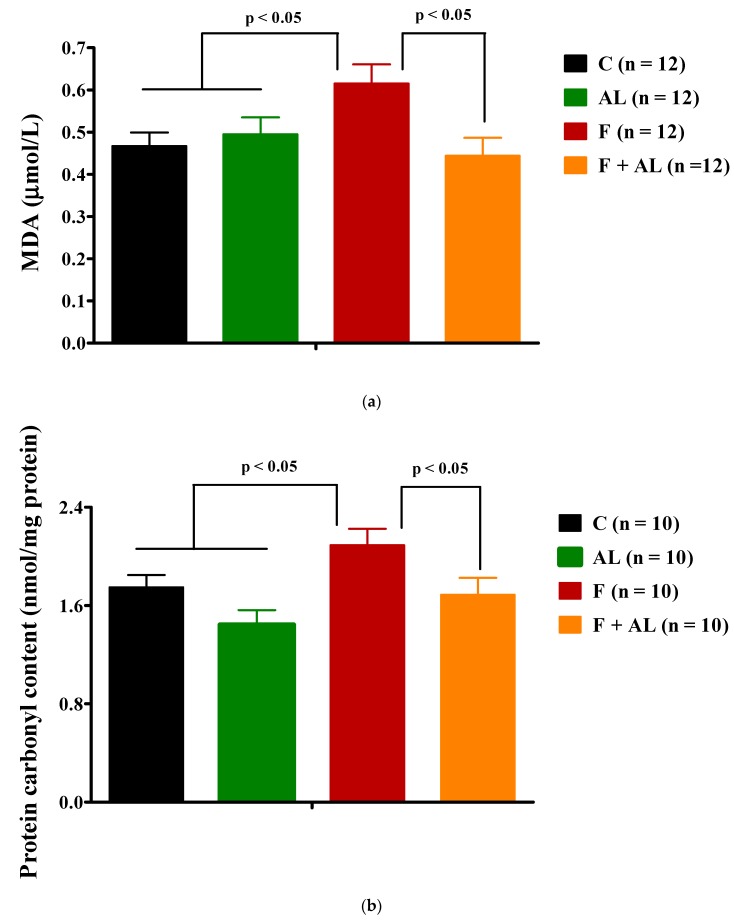

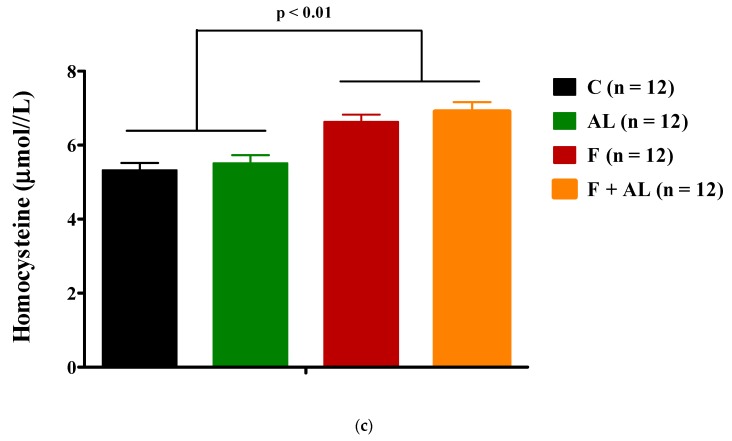
Oxidative stress markers (**a**) Plasma lipid peroxidation expressed as Malondialdehyde (MDA); (**b**) Plasma protein oxidation expressed as protein carbonyl content; (**c**) Homocysteine level, determined 12 weeks after diet and treatment in the control group (C), the alpha-lipoic acid-treated group (AL), the fructose-fed group (F), and the fructose-fed group treated with alpha-lipoic acid (F + AL).

As shown in Figure 5, plasma MDA and the protein carbonyl content were higher in the fructose group than the values recorded in the groups fed with regular chow. Also, in fructose-fed rats, the lipid and protein oxidation was significantly prevented by the chronic discontinuous treatment with AL. 

#### 3.3.2. Homocysteine

We noted that fructose feeding induced a hyperhomocysteinemia, which was not influenced by our chronic discontinuous treatment with alpha-lipoic acid (Figure 5c).

#### 3.3.3. Circulating Antioxidants Levels

##### Glutathione

Results obtained (Table 3) show that chronic fructose intake (F group) induced a decrease of total blood glutathione, reduced glutathione, and GSH/GSSG ratio compared to the plasma values of the same recorded parameters in rats fed with regular chow diet (control group). The alpha-lipoic acid discontinuous treatment was able to prevent this effect of the fructose (F + AL group) and to increase the reduced glutathione level and GSH/GSSG ratio. Furthermore, when it was associated with regular chow diet (AL group), the lipoic acid enhanced all glutathione forms, but without influencing the GSH/GSSG ratio (Table 3).

##### Glutathione Peroxidase Activity

GPx activity was lower in the fructose group as compared to the control group, and lipoic acid discontinuous treatment has succeeded in preventing GPx activity decreasing in fructose-fed rats (Table 3).

### 3.4. Histopathological Analysis

**Figure 6:**
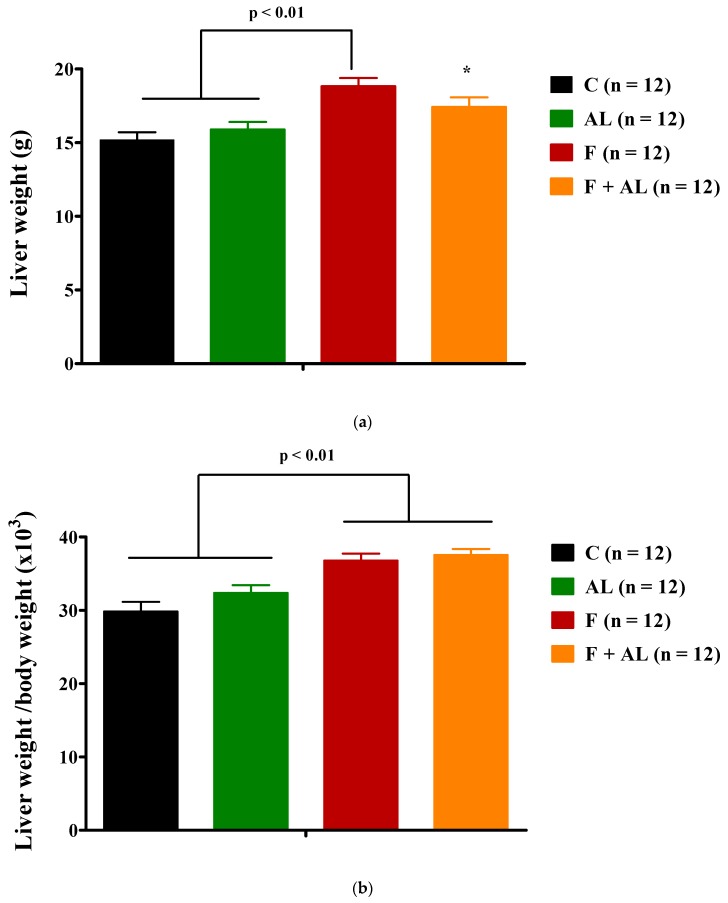
Liver weight (**a**) and Liver to body weight ratio (**b**) determined 12 weeks after diet and treatment in the control group (C), the alpha-lipoic acid-treated group (AL), the fructose-fed group (F), and the fructose-fed group treated with alpha-lipoic acid (F + AL). ^*^ *p* < 0.05: F + AL vs. C.

Fructose induced a significant increase in liver weight as compared to that of control or AL groups (Figure 6a). Liver to body weight ratio was significantly (*p* < 0.01) increased in both groups of rats fed with fructose: F group and F + AL group (Figure 6b).

**Figure 7:**
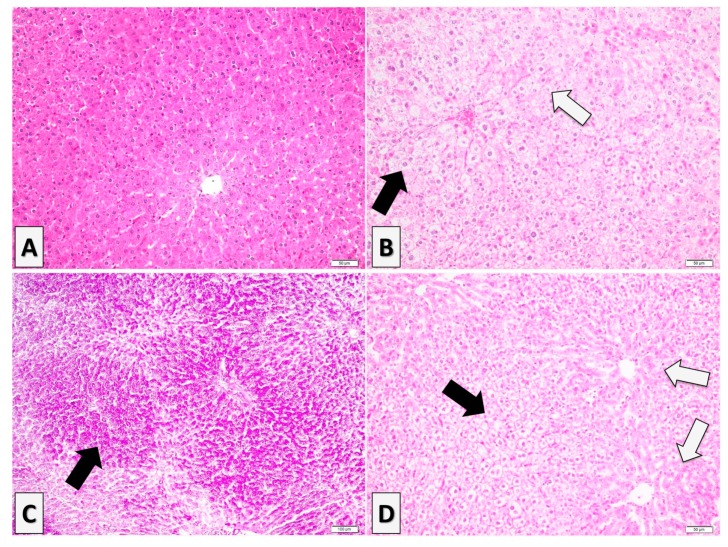
Histological features of the liver in the experimental groups: (**A**) Control group—a normal histological aspect of the liver, H&E stain. (**B**) Fructose group—diffuse degenerative changes represented by a vacuolated aspect of the hepatocytes (black arrow) with karyopyknosis and nuclear fading (blank arrow), H&E stain. (**C**) Fructose group—intrahepatocytic vacuoles display a PAS-positive material suggesting glycogen accumulation (black arrow), PAS stain. (**D**) Fructose-fed group treated with alpha-lipoic acid (F + AL group)—perilobular location of the multivacuolated hepatocytes (black arrow), with no significant changes of the hepatocytes from the centrilobular and mediolobular zones (blank arrows).

Individuals from control (*n* = 4) and lipoic acid-treated groups (AL group, *n* = 3) showed a normal histological aspect of the liver (Figure 7A). However, the liver samples collected from the fructose-fed rats (F group, *n* = 5) presented advanced and diffuse degenerative changes represented by a vacuolated aspect of the hepatocytes (Figure 7B), chiefly multiple uneven cytoplasmic vacuoles, in all the areas of the hepatic lobules (i.e., perilobular, mediolobular, and centrilobular). The nucleus of the vacuolated hepatocytes has a central or paracentral location. The multivacuolated hepatocytes exhibit some nuclear changes such as karyopyknosis or nuclear fading (Figure 7B), which may suggest cellular necrosis. The intrahepatocytic vacuoles display a PAS-positive material suggesting glycogen accumulation rather than hydropic change or lipidic inclusions (Figure 7C). In the case of liver samples collected from the fructose-fed group treated with alpha-lipoic acid (F + AL group, *n* = 5), the vacuolization of hepatocytes had a perilobular distribution, multivacuolated hepatocytes displaying similar features with the ones detected in the fructose group (e.g., multivacuolated PAS-positive aspect of the hepatocytic cytoplasm, karyopyknosis, or nuclear fading; Figure 7D). In this group, the hepatocytes located in the centrilobular and mediolobular zones did not present cytoplasmic vacuolization (Figure 7D).

## 4. Discussion

In our experimental conditions, the fructose-enriched diet induced metabolic disorders and increase in systolic blood pressure, both accompanied by hyperuricemia, impairment of hepatic function, associated with the development of pro-oxidant and pro-inflammatory status in plasma. A chronic discontinuous treatment with AL prevented and attenuated the fructose-inducing disturbances, confirming its pleiotropic effects and its direct and indirect involvement in multiple signaling pathways.

Although the effect of fructose on weight-gain has often been reported in human studies [28,29,30], it is not always obvious in the animal experimental studies [14,31]. In our experimental conditions, 12 weeks of fructose intake led to IR without influencing the body weight of rats. Thus, we obtained an experimental model of IR specific for normal weight or lean subjects. However, a decrease in the body weight of rats was observed at the end of AL discontinuous treatment in both animal groups (AL, F + AL) treated, but without being influencing the food intake. Some effect of AL on the body weight was reported when it was administered over a significant period of time (longer than 4 weeks), as dietary supplement in the food of rats [21,22,32]. It may be possible that the AL anti-obesity effect is highlighted after a high dose or in overweight context [32,33]. In our case, the reduction in the rats’ body weight under AL discontinuous treatment could rather be due to the increase in energy expenditure, especially as AL is an important cofactor of mitochondrial enzymes involved in ATP synthesis [21,34].

As previously described in other studies [31,35], the chronic fructose consumption induces an early increase in systolic blood pressure. The human and animal studies have noted a strong inverse correlation between IR and NO bioavailability [1,36] with the involvement of many signaling pathways. Thus, endothelial dysfunction with the impairment of NO-dependent vasodilatation can be induced by hyperinsulinemia, excessive ROS and RNS (reactive nitrogen species) production, and it could be amplified by hyperuricemia, inflammation, or activation of the renin–angiotensin system ‘RAS’ [37,38,39,40]. In this regard Korandji C et al. reported a rise in the vascular nitrotyrosine residues as early as the first week of fructose intake and a reduction in l-arginine/ADMA (asymmetric dimethylarginine) ratio, an index of NO bioavailability, from the second week of fructose consumption [31]. Due to its antioxidants and insulin mimetic properties, AL is able to improve NO bioavailability [41,42] and prevent the increase in systolic blood pressure, thus offering an indirect antihypertensive effect. In our study, the reduction of systolic blood pressure (F + AL group) was noted beginning with the first sequence of treatment and it was maintained through the duration of the study.

All the metabolic disorders induced by fructose (hyperglycemia, hyperinsulinemia, dyslipidemia) can be associated with its hepatic metabolism. Part of the fructose is converted into glucose through the gluconeogenic pathway, while another important part is oriented towards hepatic de novo lipogenesis [28,40,43], the fructose being more lipogenic than glucose [44]. In this sense, it was noted that long-term fructose feeding induces the expression of ChREBP (carbohydrate responsive element binding protein) and SREBP-1c (sterol regulatory element binding protein-1c), two hepatic transcription factors which control the expression of key lipogenic enzymes required for the endogenous synthesis of cholesterol, fatty acids, triacylglycerols, and phospholipids [45,46,47]. The typical dyslipidemia induced by fructose is hypertriglyceridemia that results from hepatic and intestinal TG overproduction, as well as from the reduction of TG clearance [28,47]. On the other hand, a high level of plasma and tissue triglycerides disrupts the early steps of insulin signaling pathway, like phosphorylation of insulin receptor and IRS-1 (insulin receptor substrate 1), decreases the tissue glucose uptake, and favors the occurrence of IR [40,44]. Additional intracellular glucose uptake is altered by the impairment of skeletal muscle NO-dependent vasodilation [39,40]. Unlike glucose, fructose does not stimulate insulin secretion. However, a chronic exposure to fructose can indirectly induce hyperinsulinemia as a consecutive effect of hyperglycemia and of insulin’s reduced efficacy in peripheral tissues [14,44]. In the same way, the effect of fructose on plasma lipoprotein profile is exacerbated in insulin-resistance conditions [29]. By modulation of cellular redox status, AL presents insulin-mimetic properties, induces the translocation and activation (increase of intrinsic activity) of the intracellular glucose transporter GLUT-4 [34,48] thus reducing the glycaemia, the plasma concentration of insulin as well as the HOMA-IR in fructose-fed rats [17,48]. Due to the increase in tissue glucose uptake, the key enzymes responsible for lipid metabolism can be efficiently regulated [23,47] and, consequently, the plasma triglycerides, total cholesterol and LDL-cholesterol are reduced by AL treatment.

Another important aspect of fructose metabolism is the significant depletion of hepatic ATP with an increase in uric acid synthesis [49,50]. It has been reported a dual role of high serum uric acid level: on one side, it is a consequence of IR or hypertension; however, on the other side, it could be implicated in the development of IR and cardiovascular diseases, being considered an independent risk factor for the cardiometabolic syndrome [51,52,53]. In addition, hyperinsulinemia associated to IR decreases renal clearance of uric acid [39,54] thus amplifying fructose-induced hyperuricemia [38,52,53]. In our study, the mild hyperuricemia induced by 12 weeks of fructose consumption was prevented by the chronic discontinuous treatment with AL, possibly by its insulin-mimetic properties and by its indirect effect on vascular tonus, aspects that otherwise can lead to an increase in renal clearance of uric acid. On the other hand, a reduction in the serum uric acid level, even in the AL group, could confirm an adaptive synthesis of this endogenous antioxidant according to the general oxidative status. Some small clinical trials have reported a positive correlation between the decrease in serum uric-acid levels and the improvement of the metabolic, cardiovascular, and renal parameters [49,51]. Even if a treatment for asymptomatic hyperuricemia is lacking today, larger randomized trials need to be performed where AL could be a promising candidate.

In this general context, hepatic overload could be highlighted by the increase in serum transaminase activities and by a vacuolated aspect of the hepatocytes in all the areas of the hepatic lobules. A high serum ALAT activity is considered a sensitive indicator, even for minor hepatic lesions. It is noted that fructose induces more liver injury than equivalent amount of glucose [45]. The AL discontinuous treatment was able to keep at low level plasma transaminase activities, showing a hepatoprotective effect in fructose-fed rats (F + AL group) but also in the rats fed with standard diet (AL group). Also, in F + AL group, it limited the vacuolization of hepatocytes at the perilobular area. An important part of absorbed AL is stored as lipollysine in kidneys, heart, and liver [18,41], which could explain its positive effects on serum transaminase activities and uric acid level, in both AL and F + AL groups. Nevertheless, there are some studies which mentioned rather an increase in serum ALAT or glutamate dehydrogenase activities under a treatment with alfa-lipoic acid [22,55]. This can underline that the hepatic effect of AL, even an adaptive effect of the liver at the treatment [55], is significantly influenced by the dose used and the duration of treatment, possibly like in the case of AL’s effect on body weight.

Concerning the assessment of plasma oxidative stress, the methods used in our study were various, being highlighted both oxidative damage markers (MDA and protein carbonyl content) as well the status of some endogenous antioxidant systems (glutathione and GPx activity). In accordance with other studies [14,56], we noted that 12 weeks of fructose intake induced an increase in plasma lipid and protein oxidation with a concomitant decrease in GSH/GSSG ratio and GPx activity. Oxidative stress (OS) could be an early event associated with the fructose intake, responsible for IR and endothelial dysfunction and the factor that subsequently amplifies the cardiometabolic disturbances [6,8]. An excessive and prolonged synthesis of ROS exceeds the endogen antioxidant capacity with the decline in the activity of antioxidant systems [57]. The general oxidative stress and the cardiometabolic risk could also be confirmed by the increase in total serum homocysteine concentration [58,59]. ROS accumulation could reduce Hcy re-methylation to methionine, resulting in hyperhomocysteinemia, by triggering folate depletion (methionine synthase cofactor) secondary to oxidative split of the pterinic nucleus, or by directly inactivating of methionine synthase [59,60]. AL discontinuous treatment associated with the fructose-enriched diet was able to decrease lipid and protein end-products’ oxidation, to restore the GSH/GSSG ratio and GPx activity, but without influencing homocysteine level. Concerning a potential link between AL and hyperhomocysteine and GSH, it was noted that AL catabolism involves thiol groups’ methylation, thus leading to the intensification of methionine transformation into Hcy. However, on the other hand, AL increases the Hcy transsulfuration pathway with cysteine formation and, consequently, the GSH synthesis [61]. In our study, AL had a positive influence on the reduced glutathione even in association with the standard diet.

The low grade of the inflammation developed in the fructose group and expressed by a high level of plasma CRP, a powerful indicator of inflammation and a predictor for cardiovascular diseases [35,62], can be multifactorial. Thus, OS and hyperuricemia activate various signaling pathways, such as expression of nuclear transcription factor NF-kB responsible for the synthesis of adhesion molecules and pro-inflammatory cytokines, and they could be the main ‘actors’ responsible for systemic inflammatory state [15,49]. On the other hand, inflammatory processes are involved in the development and progression of endothelial dysfunction and diabetic complications [35]. Due to its ability to reduce OS and serum uric acid level, to improve general antioxidant capacity by increasing GSH level and GPx activity or to down-regulate NF-kB [42,63] and upregulate heme-oxygenase-1 expressions [41], the AL discontinuous treatment ameliorates the elevation of serum CRP, thus preventing the systemic inflammatory process induced by chronic fructose consumption. Even if there are studies that support a decrease in inflammatory markers under a treatment with AL in humans, the available data in this context of inflammation are quite controversial, probably due to the different characteristics of population and the variety of studies design [64,65].

It is not easy to evaluate the pharmacological effects of an antioxidant, but the present study highlighted the efficacy of a chronic discontinuous treatment with lipoic acid once the metabolic disturbances induced by the fructose feeding had been installed in the rats. AL, when absorbed, has a very short half-life (30–40 min after oral and 12 min after intravenous administrations) [66], being rapidly intracellularly uptaken, metabolized, and renally excreted [19,67]. In this context, the benefits of our chronic discontinuous treatment may be partially assigned to dihydrolipoic acid (DHLA), the intracellular active metabolite of AL, which can recycle for a while the AL/DHLA system [17,67,68]. Another aspect worth mentioning is that it is difficult to correlate the pharmacokinetics (PK) and the pharmacodynamics of AL, the therapeutic effects depending especially on Cmax and AUC (the area under the curve) values, rather than the time necessary to reach maximum concentration [69]. Although, in the last decades, the beneficial effects of AL were more based on its antioxidant properties, now there is an increasing emphasis on its ability to intervene and regulate certain signaling pathways, thus exceeding the status of antioxidant molecule. As modulator of cellular redox status, AL/DHLA system influences some transcription factors and cellular signaling pathways [19,63] with positive consequences in systolic blood pressure, glucose, or lipid metabolisms and inflammatory processes.

It is true that a chronic discontinuous treatment requires further investigations and its efficacy must be supported by both plasma and tissue markers, but it may open a new perspective in improving patients’ adherence to a long-term adjuvant treatment especially in case of chronic disease and polymedication. Because the bioavailability of AL is influenced by aliments, a parenteral administration seems to be more appropriate to test the efficacy of a discontinuous treatment.

A limitation of our study could be the lack of some plasmatic pro-inflammatory cytokines (IL-6, TNF-α). These tests could have confirmed and strengthened the presence of the general proinflammatory status induced by the fructose intake, and the anti-inflammatory effects of our chronic discontinuous treatment with AL.

## 5. Conclusions

The fructose-enriched diet induced the alteration of glucidic and lipid metabolism, an increase in systolic blood pressure, accompanied by a mild hepatocytolisis (an impairment in hepatic function), and a general pro-inflammatory and pro-oxidant status. AL chronic discontinuous treatment has reduced fructose-induced disturbances, confirming its antioxidant and pleiotropic properties. Due to its positive effect on glutathione, AL treatment could be more exploited in elderly people and in pathologies associated with a decrease in endogenous antioxidant status, or in relation with the metabolism of some drugs, such as acetaminophen, or of endogenous compounds (uric acid) that expend the endogenous glutathione.

A chronic discontinuous treatment with AL is much closer to the adjuvant therapy of diabetic neuropathy and gives a better patients’ adherence to the treatment of cardiometabolic disorders. In this field, there is considerable evidence that oxidative stress plays an important role in the etiology of diabetic complications. Our study supports the promising potential of the chronic-discontinuous treatment of AL and highlights the pleiotropic effects of this antioxidant substance in metabolic disorders, such as diabetes mellitus.

## Figures and Tables

**Table 1:** Body weight, glycemia, insulin, and insulin resistance expressed as HOMA-IR assessed after 12 weeks of study.

Groups	Body Weight (g)	Glycemia (mg/dL)	Insulin (µU/mL)	HOMA-IR
C (*n* = 11)	510.57 ± 10.50	100.82 ± 1.30	50.95 ± 6.24	12.74 ± 1.57
AL (*n* = 10)	480.08 ± 9.55 ^†^	97.20 ± 3.49	54.15 ± 5.66	12.84 ± 1.23
F (*n* = 12)	515.80 ± 15.95	133.23 ± 2.5 ^***^	75.71 ± 3.74 ^***^	25.00 ± 1.47 ^***^
F + AL (*n* = 10)	465.54 ± 18.35 ^£^	122.78 ± 1.97 ^***££^	62.73 ± 3.27 ^£^	19.11 ± 1.26 ^** £^

C: Control group, AL: alpha-lipoic acid-treated group, F: fructose-fed group, F + AL: fructose-fed group treated with alpha-lipoic acid. ^†^ *p* < 0.05: AL vs. C; ^**^ *p* < 0.01, ^***^ *p* < 0.001: F and F + AL vs. C and AL; ^£^ *p* < 0.05, ^££^ *p* < 0.01: F + AL vs. F.

**Table 2:** Serum lipoproteins, uric acid, and C-reactive protein assessed after 12 weeks of study.

Groups	Total Cholesterol (mg/dL)	LDL-Cholesterol (mg/dL)	Triglyceride (mg/dL)	Uric Acid (mg/dL)	C-Reactive Protein (mg/dL)
C (*n* = 12)	53.36 ± 2.87	8.08 ± 1.28	61.86 ± 4.86	1.10 ± 0.08	0.69 ± 0.05
AL (*n* = 12)	53.08 ± 3.21	8.92 ± 1.18	67.50 ± 4.26	0.72 ± 0.04 ^††^	0.70 ± 0.05
F (*n* = 12)	75.07 ± 3.51 ^***^	56.64 ± 12.68 ^***^	331.20 ± 23.15 ^***^	2.65 ± 0.12 ^***^	0.96 ± 0.05 ^**^
F + AL (*n* = 12)	65.77 ± 3.20 ^*^^£^	35.08 ± 4.7 ^** £^	151.73 ± 7.51 ^*** £££^	0.84 ±0.08 ^£££^	0.76 ±0.05 ^£^

C: control group, AL: alpha-lipoic acid-treated group, F: fructose-fed group, F + AL: fructose-fed group treated with alpha-lipoic acid. ^††^ *p* < 0.01: AL vs. C; ^*^ *p* < 0.05, ^**^ *p* < 0.01, ^***^ *p* < 0.001: F and F + AL vs. C and AL; ^£^ *p* < 0.05, ^£££^ *p* < 0.001: F + AL vs. F.

**Table 3:** Circulating antioxidants levels.

Groups	Total Glutathione (μmol/L)	GSH (μmol/L)	GSSG (μmol/L)	GSH/GSSG Ratio	GPx Activity (U/g Hb)
C (*n* = 12)	1304.56 ± 43.78	1238.18 ± 43.34	33.19 ± 3.08	41.11 ± 4.29	869.01 ± 28.78
AL (*n* = 12)	1673.43 ± 102.05 ^††^	1566.93 ± 104.13 ^†^	53.25 ± 5.66 ^††^	33.18 ± 4.05	875.29 ± 10.00
F (*n* = 12)	1015.82 ± 109.29 ^* &&&^	921.53 ± 110.44 ^** &&&^	47.15 ± 3.66 ^**^	21.33 ± 2.96 ^** &^	790.13 ± 23.51 ^*^
F + AL (*n* = 12)	1392.23 ± 55.14 ^& ££^	1338.13 ± 55.19 ^££^	27.05 ± 3.66 ^&&& £££^	54.05 ± 5.76 ^£££^	873.17 ± 21.41 ^£^

C: control group, AL: alpha-lipoic acid-treated group, F: fructose-fed group, F + AL: fructose-fed group treated with alpha-lipoic acid. ^†^ *p* < 0.05, ^††^ *p* < 0.01: AL vs. C; ^*^ *p* < 0.05, ^**^ *p* < 0.01: F and F + AL vs. C; ^&^ *p* < 0.05, ^&&&^ *p* < 0.001: F and F + AL vs. AL; ^£^ *p* < 0.05; ^££^ *p* < 0.01, ^£££^ *p* < 0.001: F + AL vs. F.

## Abbreviations

AL: alpha-lipoic acid; ALAT, alanine aminotransferase; ANOVA, analysis of variance; ASAT aspartate aminotransferase; CRP, C-reactive protein; CVDs, cardiovascular diseases; DHLA, dihydrolipoic acid; GPx, glutathione peroxidase; GSH, reduced glutathione; GSSG oxidized glutathione; Hcy, homocysteine; HOMA-IR, the homeostasis model assessment of insulin resistance; HR, heart rate; i.p., intraperitoneal; IR, insulin resistance; LDL-C, low-density lipoprotein cholesterol; MDA, malondialdehyde; MS, metabolic syndrome; O_2_•^−^ superoxide anion; OS, oxidative stress; RAS, renin-angiotensin system; RNS, reactive nitrogen species; ROS, reactive oxygen species; SBP, systolic blood pressure; TG, triglycerides; W, week.
